# Association of M55L and Q192R polymorphisms of paraoxonase 1 gene *(PON1)* with recurrent pregnancy loss risk: A case–control study

**DOI:** 10.18502/ijrm.v19i6.9377

**Published:** 2021-07-27

**Authors:** Mehdi Alizadeh, Mahboobeh Nasiri, Morteza Samadi, Nasrin Ghasemi, Ali Moradi

**Affiliations:** ^1^International Campus, Shahid Sadoughi University of Medical Sciences, Yazd, Iran.; ^2^Department of Biology, Islamic Azad University, Arsanjan Branch, Arsanjan, Iran.; ^3^Department of Immunology, Faculty of Medicine, Shahid Sadoughi University of Medical Sciences and Health Services, Yazd, Iran.; ^4^Abortion Research Centre, Yazd Reproductive Sciences Institute, Shahid Sadoughi University of Medical Sciences, Yazd, Iran.; ^5^Department of Clinical Biochemistry, Faculty of Medicine, Shahid Sadoughi University of Medical Sciences and Health Services, Yazd, Iran.

**Keywords:** Pregnancy, Abortion, PON1, Polymorphism, Recurrent pregnancy loss.

## Abstract

**Background:**

Recurrent pregnancy loss (RPL) refers to the incidence of two or more abortions before the first half of pregnancy. Oxidative stress has been hypothesized to play a central role in RPL.

**Objective:**

To investigate the relationship between Q192R and L55M polymorphisms of *PON1* as antioxidant enzyme and the risk of RPL.

**Materials and Methods:**

In this case–control study, 110 women with RPL (case) and 110 healthy fertile women (control) referred to the Research and Clinical Center for Infertility, Shiraz, Iran were enrolled. Genomic DNA was extracted from the peripheral blood in all participants. Polymorphisms were genotyped by polymerase chain reaction-restriction fragment length polymorphism method.

**Results:**

Statistical analysis of Q192R polymorphism showed a significant difference for the RR genotype between the case and control group (OR = 11, CI = 1.39–86.87, p = 0.005) but none for the QR and QQ genotypes. No significant association was observed between the R and Q allelic frequency in the RPL participants compared to the control group (p = 0.53). Also, statistical analysis of the L55M polymorphism for MM genotype in the case group compared with the control group showed a significant difference (OR = 3.59, CI = 0.97–13.30, p = 0.042), but none for the LM and LL genotypes.

**Conclusion:**

The findings showed a significant correlation between the Q192R polymorphisms and the L55M *PON1* enzyme and RPL in this study population.

## 1. Introduction

Human reproduction is low enough so that 30% of fertilizers do not cause fetal development and abortion (1, 2). Recurrent pregnancy loss (RPL) refers to the loss of the fetus before the 20${}^{\mathrm{th}}$ day of pregnancy, which might be repeated twice or more, and are divided based on the time of abortion, abnormal preclinical abortions, and clinical abortions. In general, about 5% of couples struggle with this problem (2). It can hardly be described as a failure factor in reproduction because these problems may range from genetic causes to physiological events that occur either in the parent or in the fetus, and each of them has a cellular or particular molecular mechanisms. Therefore, genetic, environmental, immunological, hormone, infectious, and anatomical coagulation disorders are effective in its occurrence (3).

Oxidative stress and reduction of body antioxidant capacity are among the studies that might play an important role in pregnancy-related disorders such as RPL (4–6). Biomarkers of oxidative stress that cause membrane degradation, such as lipid peroxidation products, increase before an abortion, which might lead to fatal injury and degeneration of syncytiotrophoblast in early pregnancy (7, 8). Many studies have shown a significant reduction in the activity of antioxidants such as catalase (CAT) and glutathione peroxidase (GPX), and the level of selenium, as well as increased levels of malondialdehyde and lipid peroxidase in serum and embryonic tissue of RPL participants (8–10). The researchers found that the levels of antioxidants in healthy pregnant women are lower than nonpregnant women, while they are much less in women with RPL than nonpregnant women. Also, reactive oxygen species was higher in pregnant and RPL women than nonpregnant women (11).

The human body has a large number of free radical inhibition systems, including *PON1*, GPX, CAT and Superoxide dismutase. *PON1* is linked to high-density lipoprotein and is responsible for detoxification of organophosphoric compounds and neutralization of free radicals soluble in lipid peroxidation lipids (12). The gene family of paraoxonase in mammals contains three genes of *PON1*, *PON2*, and *PON3* (13). Antioxidant activity has been reported in the product of all three genes, but the most significant antioxidant role is related to *PON1* (14). The two major polymorphisms in the *PON1* gene-encoding region are Q192R (rs662) (replacing glutamine instead of arginine) and L55M (rs854560) (replacing leucine instead of methionine) (15).

*PON1* is a protein with a molecular weight of 45–43 kDa and contains 345 amino acids (14). The main and important action of *PON1* enzyme is the neutralization of free radicals and the suppression of oxidative stresses (16, 17). This enzyme together with other enzymes and antioxidants in the body creates a strong protective barrier against oxidizing substances. According to previous studies, infertility disorders such as RPL are affected by oxidative stress, and since the *PON1* is known as an antioxidant, it could play a central role in inhibiting RPL (4–6).

For the first time, this study examines the relationship between Q192R and M55L polymorphisms and the risk of women's RPL.

## 2. Materials and Methods

### Participants

This investigation was carried out as a case–control study on two different groups of women referred to the Research and Clinical Center for Infertility in Shiraz, Iran. While the control group consisted of 110 pos-menopausal women with no history of abortion and having at least one child, the case group included 110 women with RPL (two or more miscarriages) with at least a one-month gap since their last abortion. The participants were aged between 17–85 yr. (Table I).

Primary RPL was experienced by the total sample. Further tests, as well as analyses, were aimed at excluding suspected etiologies of abortion recurrence: karyotype testing of parents, hysteroscopic or hystosalpingographic inspections, antiphospholipid antibodies (consisting of anticardiolipin antibodies along with lupus anticoagulant from IgM and IgG classes), anti-thyroglobulin and anti-thyroid peroxidase, as well as hormone-related problems.

When enrolling in the experiment, tests were taken using 5-ml peripheral blood, after which the storage of the samples was performed in a tube covered with anticoagulant EDTA.

### Genetic analysis

For genotype analysis, genomic DNA was extracted from the whole blood sample using a salting-out method described by Miller (18). The Q192R polymorphism was amplified using: forward 5'-TATTGTTGCTGTGGGACCTGAG-3' and reverse 5'-CCTGAGAATCTGAGTAAATCCACT-3' primers. The L55M polymorphism was amplified using 5'- CCTGCAATAATATGAAACAACCTG-3' (forward) and 5'-TGAAAGACTTAAACTGCCAGTC-3' (reverse) primers (19).

Each polymerase chain reaction (PCR) was performed in a 25-μl tube containing 2 μl of DNA, 1 μl of each primer, 12.5 μl master mix (Amplicon, Denmark), and 8.5-μl distilled water. The PCR cycling conditions for *PON1 *Q192R consisted of an initial denaturation at 94°C for 5 min, followed by 30 cycles of denaturation for 1 min at 94°C, annealing for 1 min at 62°C and extension for 1 min at 72°C (Biorad, ABI, Foster City, CA, USA).

A final extension was carried out for 5 min at 72°C. The PCR cycling conditions for *PON1* L52M was similar to the above, except for the annealing temperature at 58°C. Restriction fragment length polymorphism (RFLP) analyses were used following the PCR amplification to detect single-nucleotide polymorphism (SNP). Digestion of the PCR products was performed at temperatures of 55°C and 37°C over a 16-hr interval with the use of BspPI (catalog number of ER1321, Fermentas) as well as Hin1II (catalog number of ER1831, Fermentas) for *PON1*-Q192R and L55M, respectively.

Electrophoresis in 3% Agarose gels was used to separate the fragments after digestion, and then staining with green viewer was applied for detection.

**Figure 1:**
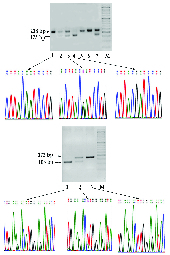
*PON1* polymorphisms electrophoretic pattern using RFLP analysis and the relative genotypes identified through Sanger sequencing. RFLP led to the detection of three different patterns observed on the gel. (A) Genotype examination of *PON1* QR polymorphism, M: Marker (50bp ladder); lanes 1, 3, and 4 relative to the QQ (AA) (238bp, QR (AG) (238, 175, 63bp), and RR (GG) genotypes (175, 63bp), respectively. It is not possible to observe 63-bp fragments on the gel. (B) Genotype testing of *PON1* LM polymorphism, M: Marker (50bp ladder), lanes 1, 2, and 3 relative to the MM (AA) (103, 69bp), LM (TA) (172, 103, 69bp), and LL (TT) (172bp) genotypes, respectively. It is not possible to observe 69bp fragments on the gel.

As shown in Figure 1, three different electrophoretic patterns were obtained RFLP analysis. Sanger method was followed to purify and sequence-relative PCR fragments for every pattern using forward and reverse primers with the use of an automated sequencer (ABI3500; Applied Biosystems). A gel visualization system (Syngene GBOX Gel Documentation 680X) was used to observe the fragments.

### Ethical considerations

This study was approved by the Ethical Committee of Shahid Sadoughi University of Medical Sciences, Yazd (Code: IR.SSU.MEDICINE.REC.1395.292). The study points were explained to the participants prior to the study, and informed consent was obtained from all.

### Statistical analysis

SPSS16 (Statistical Package for the Social Sciences, SPSS, Inc., Chicago, IL, USA) was used to analyze data statistically. Chi-square test was applied to calculate goodness of fit for the genotypes determined by observations and estimations as well as allele frequencies.

The calculation of odds ratio was performed at confidence intervals of 95%. P-values of < 0.05 indicated the significance of the differences. Evaluation of SNPs was carried out regarding deviations from Hardy–Weinberg Equilibrium with the use of Chi-square tests.

## 3. Results

In the present study, we evaluated two types of polymorphisms in the *PON1* genes in women with a history of at least two or three miscarriages compared with women with no history of spontaneous abortion (Table I). Table II shows the genotype distribution and allelic frequency of *PON1* gene polymorphisms in case and healthy women. The genotype distributions of all polymorphisms in cases and controls were in Hardy–Weinberg equilibrium.

Data analyses indicated a relationship between the RR (OR = 11, 95% CI = 1.393–86.870, p = 0.005) and MM (OR = 3.593, 95% CI = 0.9720–13.280, p = 0.042) homozygous genotypes and the risk of RPL, while no association was found between other genotypes and developing risk for RPL (Table II).

**Table 1:** Demographic characteristics of the case and control groups


**Groups**	**No.**	**Age (yr) (range)**	**BMI (kg/m ${}^{2}$)**	**Gestation age at the time of miscarriage (wk)**	**No. of abortion (%) [range]**	**No. of pregnancy (range)**
**RPL**	110	35.6 ± 12.6 (17–85)	25.5 ± 2.9	10.42 ± 4.32	= 2 (> 18.1%) [2–10] > 3 (81.9%)	0
**Control**	110	57.5 ± 6.4 (43–79)	26.9 ± 3.4	0	0	2.43 ± 0.32 (> 2)
Data presented as Mean ± SEM						

**Table 2:** Distribution of *PON1* L55M allele and genotype frequencies in RPL participants


	**Case (N = 110)**	**Control (N = 110)**	**OR**	**CI (95%)**	**P-value**
**Q192R genotype**					
**QQ**	53 (48)	44 (44)	1.183	0.686–2.039	0.541
**QR**	46 (42)	55 (55)	0.588	0.340–1.016	0.560
**RR**	11 (10)	1 (1)	11	1.393–86.870	0.005
**Allele frequency**					
**Q**	152 (69)	143 (72)	0.875	0.574–1.334	0.532
**R**	68 (31)	56 (28)	1.142	0.749–1.741	
**L55M genotype**					
**LL**	51 (46.3)	59 (59)	0.600	0.347–1.038	0.067
**LM**	48 (43.7)	38 (38)	1.263	0.727–2.195	0.406
**MM**	11 (10)	3 (3)	3.593	0.972–13.280	0.042
**Allele frequency**					
**L**	150 (68)	156 (78)	0.604	0.389–0.937	0.023
**M**	70 (32)	44 (22)	1.655	1.067–2.566	
Data presented as n (%). P-value by Chi-square goodness-of-fit test, OR: Odds ratio, CI: Confidence interval					

## 4. Discussion

The phenomenon of RPL is a difficult and stressful problem for both doctors and couples. Generally, abortion is defined as the loss of pregnancy before the 20${}^{\mathrm{th}}$ wk, and RPL as two or more abortions before the 20${}^{\mathrm{th}}$ wk (4). In 50% of the RPL cases, no specific etiologic factor is found and as such they are categorized as idiopathic, while the remaining 50% include anatomical, immunological, genetic, endocrine, thrombophilic, oxidative stress, and environmental factors. According to previous studies, one of the predisposing factors in infertility disorders, in particular, the RPL is the presence of alleles and genes in the people which leads to clinical manifestations in the participants. One of these effects is a different expression, resulting in a different function in the enzymes involved in the antioxidant role that increases or decreases the antioxidant's power and eliminates the oxidative and antioxidant balance of the body, resulting in oxidative stress.

One of these enzymes which has an antioxidant role is the *PON1* enzyme. Based on the type of polymorphism produced in the *PON1* enzyme, it can increase or decrease the activity of this enzyme and thereby increase or decrease its antioxidant capacity. Several studies point to the role of oxidative stress in infertility disorders, including in RPLs (6, 11, 20). Therefore, to prevent this, several antioxidant defense barriers function in the body. One of these defensive factors is the *PON1* enzyme, which is linked to high-density lipoprotein and prevents LDL oxidation (21), so it can be mentioned as a possible cause in preventing RPL. This enzyme has two major polymorphisms in the 192 (Q192R) and 55 (L55M) regions (15), the antioxidant activity and power are due to these polymorphisms. The rs854560 (L55M) polymorphism is associated with an increased risk of cancer, stroke, and heart disease (22–24). The rs662 (Q192R) polymorphism is associated with several diseases, such as gestational diabetes, systemic lupus erythematosus (SLE), polycystic ovarian syndrome (PCOS), and hypertension (25–28). However, so far, no study has been done to link these polymorphisms with RPL syndrome. To the best of our knowledge, this is the first study to investigate the relationship between Q192R and L55M polymorphisms with RPL syndrome. The results of this study on Q192R polymorphism show that according to the value of p = 0.005 obtained for the RR genotype [OR = 11, p = 0.005 CI = 1.393–86.870], there is a significant relationship between the genotypic frequency of this polymorphism and the incidence of abortion in women. However, the frequency of QR and QQ genotypes was not significantly different between the case and control groups. These results suggest that individuals with RR genotype are more likely to have RPL. The results also showed an L55M polymorphism for the genotype MM [OR = 3.593, CI = 0.972–13.280, p = 0.042], there is a meaningful relationship between the control and case groups regarding the MM genotype, these results suggest that individuals with MM genotype are at a higher risk of RPL. Also, the allele frequency of M and L in the control group was significant (p = 0.023). In a 2015 study on infertility of Iranian women in relation to the *PON1* enzyme at the Isfahan University of Medical Sciences, Iran, which was performed on 55 women with endometriosis and 65 women with PCOS, it was shown that individuals with MM genotype might be predisposed to infertility, while heterozygous (LM) might contribute to protecting the individual against infertility (p < 0.05) (29). The results of this study on the incidence of RPL confirmed these results.

Another Iranian study on 120 infertile men, in which the relationship between Q192R polymorphism and male idiopathic infertility was investigated, showed that there was a significant relationship between the genotypic frequency of the participants in the case and control groups (p = 0.0001); their findings showed that individuals with QR and RR genotypes are less likely to have infertility. They showed that Q192R polymorphisms of *PON1* gene are associated with a reduction in the risk of male idiopathic infertility (30), which are not consistent with our results on the RPL in women. One of the reasons for this can be the difference in the type of population in this study, as they examined male infertility. A study of 482 women with PCOS in Q192R and L55M polymorphisms found a significant correlation between the L55M polymorphism and PCOS, but this relationship was not observed for Q192R (31), The results of this study on L55M polymorphism are consistent with our study results. As mentioned earlier, the purpose of this study was to investigate the relationship between the *PON1* polymorphisms with RPL, in other words, to investigate the effects of oxidative stress caused by different polymorphisms in this enzyme. In this regard, several studies have been conducted on the effects of oxidative stress on infertility.

Ghneim and colleagues during their study estimated the antioxidant levels of selenium (Se), GPX, CAT, and also malondialdehyde oxidants in plasma, total blood, and embryo tissue in three groups of women including pregnant, nonpregnant, and pregnant women with abortions (11). The researchers found that the levels of antioxidants were lower in healthy pregnant women than in the nonpregnant women, and in women with RPL it was much less than nonpregnant women. Also, reactive oxygen species was higher in pregnant women and women with RPL than nonpregnant women. The results of this study showed that there is an effective relationship between oxidative stress and infertility. Given that antioxidant activity of the *PON1* enzyme is different depending on its genetic polymorphism (11), and according to Mackness and colleagues the antioxidant power of Q192R polymorphism was QQ > QR > RR (32), so the results of our study are consistent with the results of Ghneim and colleagues (11). In other words, in our study group, RR genotype was more than the control group (polymorphisms with the lowest antioxidant power).

Another study by Agarwal and colleagues, who examined the effects of oxidative stress on women's fertility capacity, showed that oxidative stress caused various diseases and infertility disorders, such as preeclampsia and abortions during pregnancy (6). The results of our study, which showed an increase in RR and MM stress polymorphisms in women with RPL, are confirmed by the results of their study.

Mashayekhi and colleagues, in their studies of the effects of Q and R polymorphism on infertility in Iranian women, observed that there is a significant difference between Q and R genotypes between control and infertile women. They observed that the risk of infertility is potentially reduced in populations with QR genotype (30). Data from these studies showed that QR polymorphism is associated with a reduction in the risk of infertility. The results of this study confirmed our results. The only study related to our work, conducted by Ozturk and colleagues, found no significant association between the enzymatic polymorphisms of *PON1* and RPL. It should be noted that this study was a preliminary study in which the sample size was small. The results of this study were not consistent with the results of our study (33).

The results obtained in this study might have been influenced by the race of the subjects studied and the geographical distribution and the difference in sample selection criteria that should be considered. The study of other *PON1* polymorphisms is recommended in subsequent studies because it has been shown that paraoxonase gene polymorphisms weaken or exacerbate each other's effects (34).

## 5. Conclusion

The findings of this study, which was conducted in Iran for the first time, showed that there is a clear correlation between the Q192R and L55M *PON1* enzyme polymorphisms and RPL. In the near future, with the possibility of determining the genetic map and identifying the polymorphisms of each person, the frequency and susceptibility of each person to the diseases can be understood and treated for each person specifically. Hence the polymorphism studies help us for novel treatment concepts of RPL. In general, statistical studies should be repeated in different populations. Therefore, the present study cannot conclusively prove the relationship between Q192R and L55M polymorphism with RPL until it is repeated and confirmed by others. In the present study, *PON1* polymorphisms were investigated, and it is recommended that the serum levels and activity be investigated in subsequent studies to be more conclusive.

##  Conflict of Interest

The authors declare no conflicts of interest.
